# From Provider to Advocate: The Complexities of Traumatic Brain Injury Prompt the Evolution of Provider Engagement

**DOI:** 10.3390/jcm10122598

**Published:** 2021-06-12

**Authors:** Eric Singman

**Affiliations:** Wilmer Eye Institute, Johns Hopkins Hospital, 1800 Orleans St, Baltimore, MD 21287, USA; esingma1@jhmi.edu or ericsingman@gmail.com; Tel.: +1-443-540-4105

**Keywords:** vision rehabilitation, review of systems, traumatic brain injury, concussion, patient advocacy

## Abstract

Treating a patient with traumatic brain injury requires an interdisciplinary approach because of the pervasive, profound and protean manifestations of this condition. In this review, key aspects of the medical history and review of systems will be described in order to highlight how the role of any provider must evolve to become a better patient advocate. Although this review is written from the vantage point of a vision care provider, it is hoped that patients, caregivers and providers will recognize the need for a team approach.

## 1. Introduction

As we advance our understanding of the protean manifestations of traumatic brain injury (TBI) on the visual system in particular [1,2], and the entire patient in general [3,4,5], the vision care provider (VCP) will find it difficult to remain tightly focused on the patient’s vision problems. As the VCP delves into a thorough review of systems, medications, social and medical histories, they will find that they have information that must be shared with the entire care team and that there will be times when they must serve as a patient advocate. Becoming an effective patient advocate requires skill, training and practice [6,7,8,9]. However, patients and their families clearly value advocacy on their behalf [10,11,12]. Furthermore, it is becoming clear that the optimal TBI care team of the 21st century is not only an interdisciplinary one [13,14,15,16,17,18], but this may also be the best paradigm to reduce the high societal costs of TBI [19,20,21,22]. Notably, patients and their family supporters have made it clear that they prefer and expect interdisciplinary care [23,24], so it is unlikely that a VCP will be able to avoid increasing levels of engagement with the TBI team. This review will annotate some of the more common systemic impacts of TBI and discuss pathways for the VCP who wants to maximally incorporate into the entire TBI team. It should be mentioned that what will be discussed for concussion or mild TBI is also applicable to severe TBI. Furthermore, it is hoped that this review will not only guide the VCP to a more holistic assessment of patients with TBI, but also direct them to refer to specialists should they uncover a heretofore unaddressed problem in their patients.

## 2. The Patient History

The VCP’s approach to a patient known to have suffered TBI starts with a history of the injury (or injuries). When the care delivered is in the outpatient setting weeks or months after the acute trauma (as is most often the case), the focus of the history might be on how the health status of the patient has changed after TBI as well as the patient’s trajectory of recovery. It should be noted that in mild TBI, where radiographically-identified brain damage is often absent, the VCP must explore visual functions that are more subtly disrupted by concussion.

The patient’s past medical history is also valuable because this might reveal conditions that could have an impact on the damage caused by TBI. For example, it is known that a hypercoagulable state develops after severe TBI [25,26]. Notably, thrombosis of the cerebral venous sinuses can lead to chronic intracranial hypertension, which in turn can cause sight-threatening papilledema after TBI [27]. It should be mentioned that papilledema from intracranial hypertension can occur after even mild TBI and even in the absence of venous sinus thrombosis [28]. This highlights the duty of the VCP to recognize that common complaints in patients suffering TBI, such as headache and blurred vision, overlap with those caused by other conditions.

Finally, a clear understanding of the patient’s past medical history could reveal pre-morbid conditions that could impede rehabilitation. For example, it has been reported that patients with underlying Ehlers Danlos syndrome may have slower and less complete recovery after mild TBI [29], and that pre-injury psychiatric migraine symptoms are risk factors for worse outcomes at 6-months after any level of TBI severity [30].

## 3. The Review of Systems

An opportunity for the VCP to truly familiarize themself with the patient’s real-time status comes during the review of systems (ROS). A robust ROS cannot be overemphasized when working with concussion patients because of the wide variety of health problems experienced after brain injury. One should not assume that other members of the TBI team have fully explored the ROS; the VCP may very well be the first and only provider to identify a serious concern. The following list is undoubtedly incomplete. Our goal is to highlight the complexity of medical problems faced by patients with any level of TBI and touch upon some clinical connections that should not be overlooked. The knowledge gleaned by a thorough ROS may be used to direct a patient to a specialist needed by the patient, but not yet on the care team or at least alert the patient’s primary care provider of a concern that needs to be addressed. As mentioned above, the VCP should remember that those concerns associated with mild TBI are assuredly applicable to patients with severe TBI, and should be explored and/or addressed once the patient has reached the appropriate level of improvement.

### 3.1. General Health

Weight loss [31] and weight gain [31,32,33] are common after TBI. Weight loss could be associated with insufficient calories or nutrients required to effect recovery and could signal other concerns, such as depression or financial problems. Weight gain can be associated with lack of motivation to exercise [34], fatigue [35,36,37,38], insomnia [35,39,40,41], and hypersomnia [42], all of which are known to occur after TBI. Weight gain can lead to secondary conditions that can directly impact the visual system, such as hypertension and diabetes. Patients should specifically be asked about changes in weight as well as why they believe these changes occurred.

### 3.2. Vision

The VCP will frequently be consulted when patients suffer TBI because vision problems are common in this condition. Patients might report diplopia [1,43,44], photophobia [45,46,47,48], dry eye [49,50], eye strain [51], blurred vision [2,52], visual acuity loss [53], visual field loss [2,53] and reduced color vision [53]. However, it is important to recognize that patients with TBI often cannot specifically articulate their vision complaints and the use of symptom survey questionnaires could be valuable [54,55]. Although the VCP may offer the therapeutic avenue for vision problems, it is still critical to ensure the other members of the patient’s care team are provided situational awareness, including the schedule for care. Patient’s with TBI often require multiple types of therapy and results could be unsatisfactory unless care is coordinated so as not to overburden patients.

### 3.3. Vestibular System/Auditory

Common complaints related to the ears after TBI include reduced hearing [56,57], hyperacusis [58,59], tinnitus [57,58,60,61], dizziness/vertigo [4,62] and otorrhea [63]. The VCP should be careful to ask patients whether their tinnitus is pulsatile, as this might suggest abnormal CSF pressure (high or low) or dehiscence of the semicircular canal. Both of these conditions can be associated with dizziness and can be overlooked. The VCP might be the only one to identify papilledema in patients with intracranial hypertension. Furthermore, the VCP has a perfect opportunity to look for nystagmus induced by sound (i.e., Tullio’s phenomenon), a sign of semicircular canal dehiscence [64].

### 3.4. Olfactory

Anosmia or changes in sense of smell [65] and rhinorrhea [63] can occur after TBI. It is critical to ask the patient about rhinorrhea. A past history of sinus allergies does not guarantee that the post-TBI discharge is mucous. A CSF leak should be suspected, particularly when the discharge is clear, colorless and thin; CSF will test positive for Beta-2 transferrin [66]. Patients with CSF leaks often have symptoms of CSF hypotension and are at risk for a life-threatening spontaneous subdural hematoma [67] and/or cerebral infection [68]. Urgent referral for a suspected CSF leak is therefore critical.

### 3.5. Oral

Post-traumatic oromandibular dystonia [69], often associated with bruxism, occurs after TBI. Furthermore, it has been shown that bruxism contributes not only to post-TBI headaches [70], but is also correlated with the presence of tinnitus [71]. While dentists are perhaps most likely to identify bruxism because of secondary tooth wear [72], TBI patients might unfortunately not be guided to include oral health professionals on their care team, so specifically asking patients about bruxism could reveal a treatable and impactful diagnosis.

### 3.6. Cardiovascular

Orthostatic hypotension [73] has been reported to occur after TBI and can manifest as symptoms commonly seen after TBI including dizziness, fatigue, nausea and headache. This condition can also be associated with tachycardia, and might be misinterpreted as anxiety attack; referral for a tilt-table test could resolve this situation and lead to appropriate treatment [74].

### 3.7. Respiratory

Sleep apnea [40,42] has been reported to develop in patients who incurred a TBI. This condition can be associated with bruxism although a recent systematic review suggests this is not well supported [75]. Sleep apnea can be associated with floppy eyelid syndrome [76] and it behooves the VCP to evaluate patients for this condition because it can cause irritated eyes or even cornea abrasions during sleep. Notably, an association between sleep apnea and intracranial hypertension has been suggested; although the relationship between these two conditions may not be sufficient to recommend fundus examinations on every patient with sleep apnea [77], it seems reasonable for VCPs to look for papilledema in all of their sleep apnea patients.

### 3.8. Gastrointestinal

Nausea [4,62,78] and altered appetite [79] are frequent in the acute post-TBI period. When these problems persist, it is reasonable to look for other underlying problems associated with TBI that can precipitate nausea, e.g., migraine, vestibular dysfunction and abnormal CSF hydrodynamics.

### 3.9. Genitourinary/Endocrine

Patients with any level of TBI have been reported to develop erectile dysfunction [80] and altered menstrual patterns [81,82]. These problems can add to the emotional and/or social distress that often burdens TBI patients, can interfere in family planning, and unfortunately, they are probably less likely to be explored. Recognizing these problems helps validate the difficult circumstances faced by TBI patients and also starts the path to their resolution. Notably, erectile dysfunction after TBI stems not only from psychological stressors; hypogonadism is not uncommon following even mild TBI and testosterone replacement has demonstrated value [83]. Aside from the alterations in sex hormone levels, patients with TBI can also develop hypopituitarism [80,84,85].

### 3.10. Musculoskeletal

The biomechanics of TBI are such that cervical injuries are frequent sequelae [86]. Cervical injuries cause pain [87,88] that can interfere with sleep and range of motion. Moreover, traumatic neck pain can be associated with dizziness, visual disturbances and altered balance [89], hampering visual and vestibular rehabilitation efforts. Occipital neuralgia and other forms of cervicogenic headache can refer pain to the orbit, misdirecting diagnostic and therapeutic efforts. A high level of suspicion for these conditions followed by a referral of the patient to pain management specialists for consideration of occipital nerve blocks may provide the patient with critically needed relief [90]. It is also valuable to ask patients whether they might have hypermobile joints, suggestive of underlying conditions such as Ehlers Danlos syndrome (EDS). Patients with EDS are more likely to suffer vertebral fractures [91]. In addition, brain injury may even unmask heretofore undiscovered diagnoses of hypermobile EDS [29,92]. When patients demonstrate hypermobility, preferably via a simple in-office evaluation of their Beighton score [93], a referral to a geneticist is advised.

### 3.11. Neurologic

Headache is the most common sequela of TBI of any level of severity [94,95]; this complaint not only encompasses generalized headache but also localized head pain and migraine [95]. Migraineurs may experience an increase in frequency, severity or duration of their migraines after concussion [96], and patients often experience their first migraine after TBI. Furthermore, there is a correlation with post-traumatic migraine, cognitive impairments and protracted recovery after TBI [97]. Migraine is one of the most persistent complaints after TBI, often lasting at least 1 year after injury [98] and causing reduced quality of life at 5 years post-injury [99]. Migraine is associated with an elevated risk of co-morbid conditions, including depression, anxiety and insomnia [100]; recognizing that these complaints are extremely common after TBI, one must wonder whether migraine perpetuates TBI symptoms. At any rate, referral of patients with post-concussion migraine to headache specialists who can reduce the burden of migraine is essential and it may be found that this shortens the duration and intensity of post-concussion syndrome. Notably, Chiari malformation, a congenital condition in which the cerebellar tonsils descend into the foramen magnum, and which can remain subclinical in many patients, can become symptomatic after TBI [101]. Patients with stereotypic post-TBI symptoms who are not improving should be explored for this condition by simply reviewing the brain imaging.

### 3.12. Integumentary

If the review of systems elicits a complaint of easy bruising, then further questioning of the patient and family should follow. Easy bruising can be seen with a number of conditions, including Ehlers Danlos syndrome [102] and bleeding disorders [103]. Notably these conditions may potentiate damage after TBI [29,104].

### 3.13. Hematologic

Hypercoagulability after severe TBI is well recognized and carries a risk of worse outcome [25,26,105]. Although it is unlikely that the VCP providing rehabilitative care will diagnose this problem, they should be aware that patients might be placed on anticoagulants and that could have an impact in planning ophthalmic surgical procedures.

### 3.14. Immunologic

There is strong evidence to support that concussion involves a sterile inflammation of the brain [106], as indicated by elevated levels of plasma cytokines in patients. Notably, elevated inflammatory markers are seen in patients with migraine as well [107], raising the question of whether the two conditions may perpetuate each other. It has also been shown that Mast cells degranulate after mild TBI [108,109]. It should be mentioned that there is a condition known as mast cell activation syndrome [110,111], although there does not appear to be published studies exploring whether patients with this condition have poorer outcomes after TBI. It seems reasonable to ask patients whose TBI symptoms are prolonged as to whether they might have symptoms of mast cell activation syndrome [111].

### 3.15. Infectious

There is a published report of a large number of patients with refractory post-concussion syndrome with symptoms lasting a year who secondarily tested positive for Lyme disease [112]. Although further study on this topic is clearly needed, it seems reasonable to explore a diagnosis of Lyme disease in patients with chronic post-concussion syndrome, particularly in areas where Lyme disease is endemic.

### 3.16. Psychiatric

Traumatic brain injury has been consistently demonstrated to cause dementia [113], cognitive deficits [114], anxiety and depression [115,116]. These changes can be protracted or even permanently disabling. The VCP should question every TBI patient to ensure that they have had a neuropsychological assessment. Those patients for whom this has not been scheduled should be strongly encouraged to seek such an evaluation.

Table 1 summarizes the clinical presentations described above.

## 4. Medications and Social History

There are many prescription medications offered to patients that cause side-effects mimicking complaints commonly reported after TBI, such as dizziness, somnolence or nausea. The VCP should take note of a patient’s medication and discuss with the patient whether any TBI symptoms worsened after any particular medication was initiated. A social history is also critically important to determine how a patient’s habits and social support structure might impact recovery. For example, it has been reported that perceived social support [117] and early return to exercise [118] may be salutary after mild TBI, while TBI may be a risk factor for problem gambling [119] and alcohol abuse [120]. A history of TBI is significantly more prevalent among the homeless [121] and poor [122]. It is likely that TBI causes a downward social drift, since patients often cannot work and become isolated because of their psychiatric conditions and substance abuse. VCPs and all health care providers must recognize that poverty may be the most deleterious sequela of TBI and that poverty reduces overall health and life expectancy [123]. For this reason, the VCP must determine whether the TBI patient has access to adequate resources and guide patients to social work professionals if the patients do not have such access.

## 5. The Post-Examination Conversation

The final portion of the VCP visit involves a discussion with the patient concerning the findings of the vision examination and how those findings can guide therapeutic pathways. Like every provider who treats patients with TBI, the VCP will often need to refer patients to members of the TBI-treatment team who might not already have been recruited and to facilitate communication of rehabilitative plans. The value of including a patient’s significant social supporters in these conversations cannot be overstated [124]. For patients with mild TBI, the main thrust of rehabilitation will usually concern lingering visuomotor problems. Because the VCP has explored the patient’s circumstances, such as availability of transportation, insurance coverage, tolerance for orthoptic exercises and distance to a practitioner, the VCP can help the patient determine whether vision rehabilitation should commence or be held in abeyance until other complaints are relieved, as well as how best to balance in-office and at-home vision rehabilitation venues [125,126].

## 6. Patient Advocacy

The VCP complements the team of specialties demonstrated as necessary to support TBI patients, including neurology, neuropsychology, neurosurgery, pain management, neuro-otology, dentistry, physiatry and social service professionals. Like every other member of this team, the VCP is in a unique position to clarify the nexi between patients’ complaints that are specialty-specific with those that are non-specialty-specific, and ensure these points are adequately communicated to other members of the care team and patients. The result will be that patients and their supports have the situational awareness to make optimal, informed decisions. How far the VCP goes is up to the individual provider. At the very least, every provider should ensure that their findings and suggestions are distributed to the TBI care team. Providers should also help identify and even recruit (via referral) experts that might not yet be on the TBI care team. Optimally, any provider on the care team will want to create an atmosphere of advocacy for the patient. Because TBI patients often have such complex situations, they may not be the best person to serve as their own navigator, although they must be included in every decision. When possible, guiding a family member to serve as an advocate can be helpful and there are a number of resources to help this process (for example, [127]). Finally, it must be mentioned that there are professional patient advocates who are well trained, follow a code of ethics and carry professional liability insurance; these are usually healthcare professionals who have transitioned into patient advocacy. Although these professionals might not specialize in patients with TBI, the learning curve would likely be much shorter for them should a member of the TBI team want to serve as a mentor.

## 7. Conclusions

TBI recovery can last for months or years. It is likely that the VCP will grow very familiar with patients and their supporters during this difficult period in a patient’s life. Perhaps the best mindset is that doctors, patients and anyone involved in the patient’s support should be viewed as stakeholders in the recovery process. An in-depth and broad understanding of the medical and social ramifications of TBI should encourage caregivers to actualize the value of their interactions and lead the way toward the best outcomes.

## Figures and Tables

**Table 1:** Topics to Explore when Performing the Review of Systems.

System	Possible Areas of Concern
General Health	Changes in weight, sleep, energy
Vision	Changes in acuity, field, comfort, light sensitivity, ocular motility
Vestibular/Auditory	Changes in hearing, balance, as well as dizziness, otorrhea
Olfactory	Change in smell, as well as rhinorrhea
Oral	Clenching or bruxism
Cardiovascular	Orthostatic hypotension
Respiratory	Sleep apnea
Gastrointestinal	Nausea, changes in appetite
Genital/Endocrine	Erectile dysfunction, changes in menses
Musculoskeletal	Pain (including possibly referred pain), exploring joint hypermobility
Neurologic	Headache, migraine, exploring Chiari malformation and intracranial hypertension
Integumentary	Exploring easy bruising
Hematologic	Exploring hypercoagulability
Immunologic	Exploring Mast cell activation
Infectious	Exploring Lyme disease
Psychiatric	Changes in cognition, mood

## Data Availability

Not applicable.
